# Delivering tactile stimuli via mobile browsers: A method for remote multisensory research

**DOI:** 10.3758/s13428-026-02995-1

**Published:** 2026-04-08

**Authors:** Rebecca J. Hirst, Kalvin Roberts, Martina Seveso, Alan O’Dowd, Jonathan W. Peirce, Fiona N. Newell

**Affiliations:** 1 School of Psychology and Institute of Neuroscience, Trinity College Dublin, Dublin, Ireland; 2 Open Science Tools Ltd., Nottingham, UK; 3 School of Psychology and Neuroscience, University of St. Andrews, St. Andrews, Scotland; 4 School of Psychology, University of Nottingham, Nottingham, UK

**Keywords:** Online experiments, Vibrations, Smartphone, Redundant target effect, Reaction times, Tactile perception, Multisensory integration, Stimulus timing accuracy

## Abstract

Online methods are becoming an essential part of the behavioral scientist’s toolkit. While the remote presentation of visual and auditory stimuli has been shown to be reasonably accurate (Bridges et al., 2020), less is known about the feasibility of delivering tactile stimuli via the browser. In this study, we extend remote experimentation to the tactile domain by replicating a well-established multisensory phenomenon – the redundant target effect (RTE) – using Android smartphones. The RTE, wherein response times are faster to multisensory (bimodal or trimodal) than unimodal stimuli, was robustly replicated in an online sample using a browser-based task. In addition to the behavioral replication, we evaluated the timing accuracy and precision of visual, auditory, and tactile (vibration) stimuli presented via mobile browsers. Visual and auditory stimuli exhibited small, consistent lags, while vibration stimuli showed a greater delay. Further analysis indicated that much of this delay was attributable to the gradual ramp-up of smartphone vibration motors, rather than limitations of the browser itself. These results support the feasibility of using consumer mobile devices to study tactile and multisensory processing in uncontrolled environments. The significance of the current manuscript is twofold: first, we demonstrate that scalable, at-home user studies can incorporate haptics without bespoke apps; second, we provide benchmark timing data to inform the feasibility of future research implementing tactile perception measures in remote behavioral research.

**Supplementary Information:**

The online version contains supplementary material available at 10.3758/s13428-026-02995-1.

## Intoduction

Online (web-based) research methods have become a core part of the behavioral scientist’s toolkit. The ability to launch experiments via a simple URL enables researchers to recruit larger, more diverse samples, collect data rapidly, and reduce costs (see Grootswagers, 2020, for a primer). Furthermore, web deployment reduces the need for bespoke app development, which can be a technical barrier for researchers without a computer science background. However, as with any methodology, web-based experimentation presents challenges – particularly in ensuring the accurate and precise presentation of stimuli and measurement of reaction times. These issues are well studied for visual and auditory stimuli, particularly in experiments run on desktop or laptop computers, but the viability of tactile stimulus delivery – especially via smartphone browsers – remains largely untested. In this study, we ask whether web-based smartphone-delivered vibration stimuli are suitable for behavioral research. To address this, we first attempt to replicate the redundant target effect (RTE) – a well-established perceptual phenomenon – in a mobile browser-based setting. Second, we assess the timing accuracy and precision of vibration delivery on Android smartphones. Together, these findings inform whether and how vibration stimuli can be effectively incorporated into remote experiments measuring tactile perception.

Transferring tactile research to remote testing frameworks presents unique challenges, as such studies have traditionally relied on specialized hardware to deliver stimuli directly to the skin (Killebrew et al., 2007; Woodward et al., 1990). In contrast, one of the key advantages of online research is its accessibility across a wide range of consumer devices, including laptops, tablets, and smartphones. This cross-platform flexibility enables researchers to harness web application programming interfaces (APIs) that provide access to device hardware, thereby expanding the methodological possibilities for online experimentation. For example, webcams can be used for eye tracking (Papoutsaki et al., 2016) or face detection (Levordashka et al., 2023). On smartphones, researchers can access sensors such as the gyroscope (Kuhlmann et al., 2021), and – crucially for the present study – the vibration motor, enabling basic tactile stimulation without specialized equipment.

Tactile vibration stimuli have the potential to support a wide range of online experimental paradigms. However, before pursuing complex applications, it is essential to establish whether basic, well-characterized effects can be reliably reproduced. To this end, we selected the redundant target effect (RTE) as a model paradigm to evaluate the feasibility of remotely delivering vibration stimuli. In a typical RTE task, participants respond as quickly as possible to the onset of a stimulus, which may be presented in unimodal, bimodal, or trimodal combinations of auditory, visual, and tactile signals. The RTE is observed when responses to bimodal or trimodal stimuli are faster than those to unimodal stimuli (Hershenson, 1962). Redundant target effects have been used to assess computational models underlying multisensory integration. Specifically, they have been used to compare models of sensory processing such as *Race Models* (whereby the fastest signal to be processed triggers a response) and *Coactivation Models* (whereby signals present on two channels contribute to a common pool of activation that initiates a response, Miller, 1982) – for a tutorial on testing race models in RTE paradigms see Gondan & Minakata, 2016. Of note, the focus of the current manuscript is not to assess computational models underlying multisensory response times; rather, the focus is to assess the viability of using vibration stimuli in such paradigms for remote testing of tactile perception.

Smartphones have previously been used to examine the redundant target effect (RTE), though not via web-based applications. Pomper et al. (2014) conducted a lab-based study in which participants completed an RTE task using a smartphone and custom code running natively (offline/locally) for stimulus delivery. Their findings showed faster response times in bimodal (audio-visual, audio-tactile, visuo-tactile) conditions compared to unimodal conditions, consistent with the RTE. However, they found no significant advantage for trimodal over bimodal targets.

More recently, several studies have explored the development of native apps to assess multisensory perception (including vibration-based stimuli). Inuggi et al. (2024) shared an Android application, PsySuite, developed to deliver specific multisensory psychophysics tasks involving vibration stimuli. PsySuite includes pre-created tasks, including Temporal Interval Discrimination and Double Flash Illusion paradigms and the authors share behavioral validation of the paradigms alongside measurements and comparison of stimulus durations and intervals between two Android devices. In another recent study, Roussel et al. (2025) also implemented an Android app to deliver audio-tactile stimuli; they found that looming sounds reduced tactile response times by 20–25 ms compared with static sounds. Interestingly, Roussel et al. (2025) also report a novel method for incorporating accelerometer data to enhance response-time measurement (using the acceleration change from a screen tap to indicate response onset), illustrating the usefulness of mobile device sensors for behavioral measurements. The use of native app development for studying multisensory perception has also been implemented in clinical contexts. Nunez et al. (2025) recently compared a digital health iPhone app (CatchU® v3.1.2) to an in-lab “tristimulator” setup to assess visual-somatosensory integration. They report comparable effects from app-based and in-lab methodologies; in fact, the app-based approach yielded less variable responses. Together, these studies illustrate that remote assessment of multisensory integration is possible via bespoke apps and that these have both research and clinical practical applications.

Although these existing studies herald the development of remote platforms to assess tactile perception, one challenge is that these bespoke apps typically require knowledge of specific programming languages and familiarity with app development and deployment (for example, the Kotlin programming language, a language with which researchers in psychology are not typically familiar). These steps may prove challenging and time-consuming for scientists without a computer science background to integrate into their existing test frameworks. In this manuscript, we illustrate how researchers can integrate vibration stimuli into their experiments using existing web APIs and open-source experiment builders. We use PsychoPy/Pavlovia as an example, although the same code snippet can be applied to any builder that allows custom JavaScript code insertion (e.g., jsPsych, De Leeuw et al., 2023). We hope this approach enables scientists to add vibration stimuli to their paradigms flexibly and provides baseline data on the accuracy and precision of vibration stimulus timing when delivered in this way.

Additionally, while Inuggi et al. (2024) and Roussel et al. (2025) offer valuable timing data for stimuli delivered via native apps, their measurements face challenges similar to those noted in prior studies of web-based timing (Bridges et al., 2020). Specifically, their timing of synchrony relies on using one stimulus as a reference point (e.g., measuring the lag of visual or vibration onset relative to auditory onset), which does not account for potential delays in the baseline stimulus itself. In contrast, we introduce a new method using WebSockets (a communication protocol between websites and a local server) to provide “ground truth” unimodal timing measurements (Melnikov & Fette, 2011; Roberts et al., In Prep).

The present study aimed to replicate and extend previous findings (Pomper et al., 2014) using a fully remote, browser-based testing framework. Specifically, we investigated whether the redundant target effect (RTE) could be reliably observed under real-world conditions, where uncontrolled environmental variability may introduce additional noise. In parallel, we provide practical guidance for incorporating tactile vibration stimuli into web-based experiments. Our hypotheses and analysis plan were preregistered and are publicly available via the Open Science Framework (https://osf.io/qf847/).

In addition to the behavioral replication, we present the first measurements of timing accuracy for visual, auditory, and tactile (vibration) stimuli delivered via a browser, using a novel WebSocket-based approach (Roberts et al., In Prep). With this, we aim to contribute: (a) a simple method for integrating vibration stimuli into remote experiments; (b) a technique for evaluating the temporal precision of browser-based vibration delivery; and (c) benchmark data to guide researchers on the expected timing characteristics of such stimuli across devices.

## Replicating the redundant target effect

### Method

#### Participants

We conducted a Monte Carlo power analysis using the *Superpower* package in R (Lakens & Caldwell, 2021), based on the means and standard deviations reported by Pomper et al. (2014). Using a conservative alpha level of 0.001, the analysis indicated that a minimum of 33 participants would be required to achieve 90% power. The full analysis code is available on the Open Science Framework (https://osf.io/qf847/).

Participants were recruited via Prolific (www.prolific.co) using the following inclusion criteria: residency in the USA, UK, Ireland, Australia, Canada, or New Zealand; balanced sex distribution (50/50); fluent in English; self-reported normal or corrected-to-normal vision; and no reported hearing difficulties. Participants were also asked to confirm the absence of any conditions affecting visual, auditory, or tactile functioning, as well as any photosensitive neurological conditions.

The initial sample included 38 participants. Six participants were excluded for the following reasons: one reported never hearing the auditory tone, one never saw the visual target (a white screen), one failed to respond to any unimodal visual targets, one failed to respond to any tactile targets, and two did not respond to vibration stimuli during the initial adjustment phase, which prevented progression in the task. The final sample consisted of 32 participants (mean age = 34.7 years, range = 21–53 years; 17 female).

The study was reviewed and approved by Trinity College Ethics Committee (approval number X2215).

#### Implementation

All task files can be downloaded from Pavlovia.org, shown on the OSF project page. The task was created in PsychoPy (version 2022.2.5) (Peirce et al., 2019, 2022) and hosted on Pavlovia (pavlovia.org). Vibration stimuli were implemented using a custom code component that invoked the navigator.vibrate() function at the start of each routine (coinciding with the expected onset of the visual and auditory stimulus). This function accepts either a single value (indicating the pulse duration) or an array of values (for complex vibration patterns). In our study, a single 200-ms pulse was used.

We restricted the study to Android devices running the Chrome browser. This decision was driven by our goal to demonstrate vibration delivery through a standard web browser rather than a native app. Although native applications offer more direct access to device hardware, they typically require greater development resources and expertise (the user would need to know how to deploy an app to a user’s phone). In contrast, open-source JavaScript libraries commonly used by behavioral scientists, such as PsychoPy/PsychoJS (Peirce et al., 2022), jsPsych (De Leeuw et al., 2023), and labJS (Henninger et al., 2019), allow behavioral scientists to deploy experiments more broadly and with less technical overhead. While hardware access in web apps is generally limited due to browser security constraints, vibration control is supported in certain environments – specifically Android and Chrome – making it a viable option for tactile stimulus delivery in remote behavioral research.

#### Stimuli

**Fig. 1:**
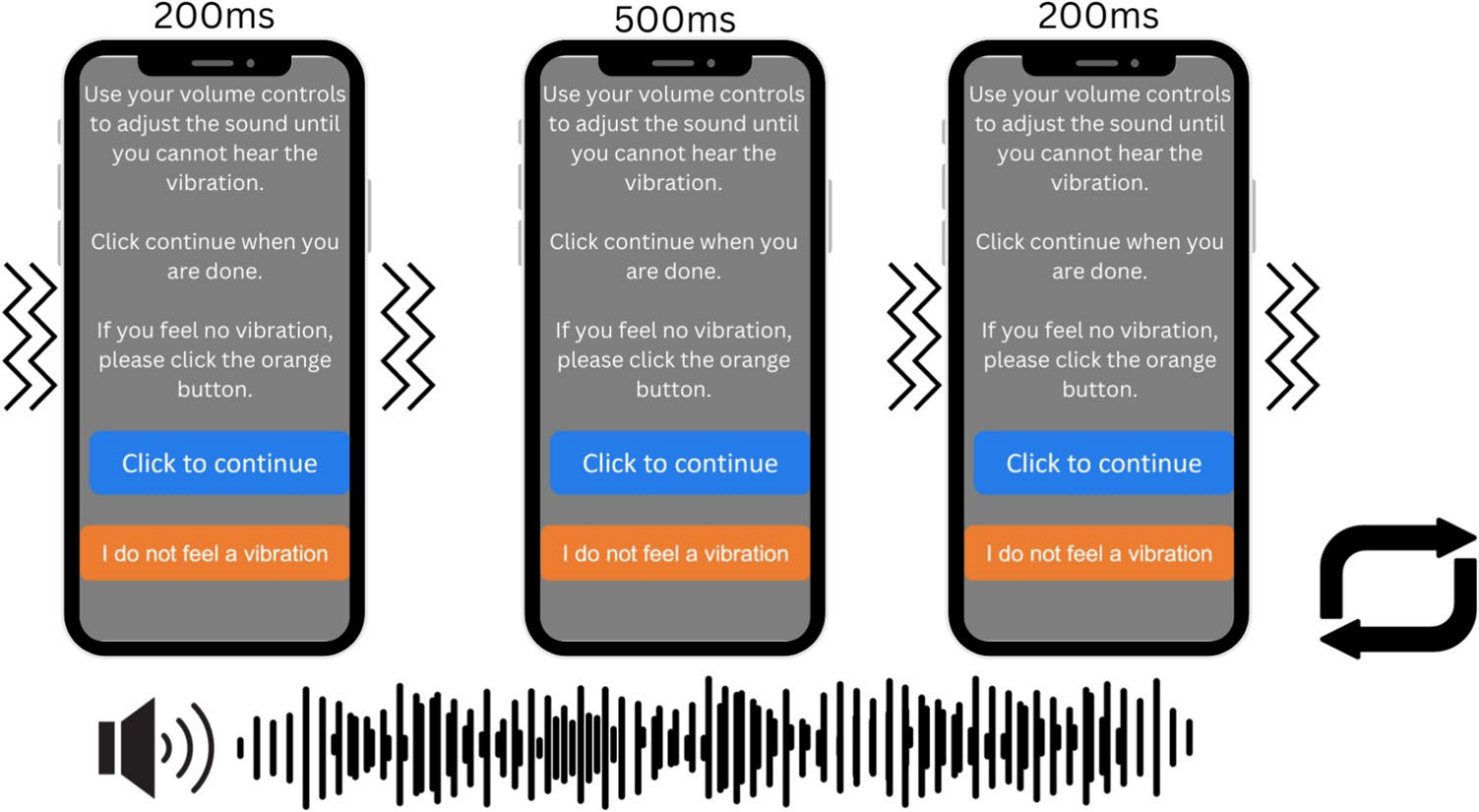
Before starting the task, participants were asked to adjust the volume of their phone until a continuous stream of brown noise masked the sound of a vibrating pulse. Participants were presented with white text on a mid-gray background which read “Use your volume controls to adjust the sound until you cannot hear the vibration. Click continue when you are done. If you feel no vibration please click the orange button” beneath this there were two buttons, a blue button with the text “Click to continue” and an orange button with the text “I do not feel a vibration”. The pulse was presented on a cycle of 200-ms pulses followed by a 500-ms inter-stimulus-interval and the brown noise was presented continuously until the participant pressed “continue”. If the participant indicated that they did not feel a vibration, then the rest of the task was not presented

**Fig. 2:**
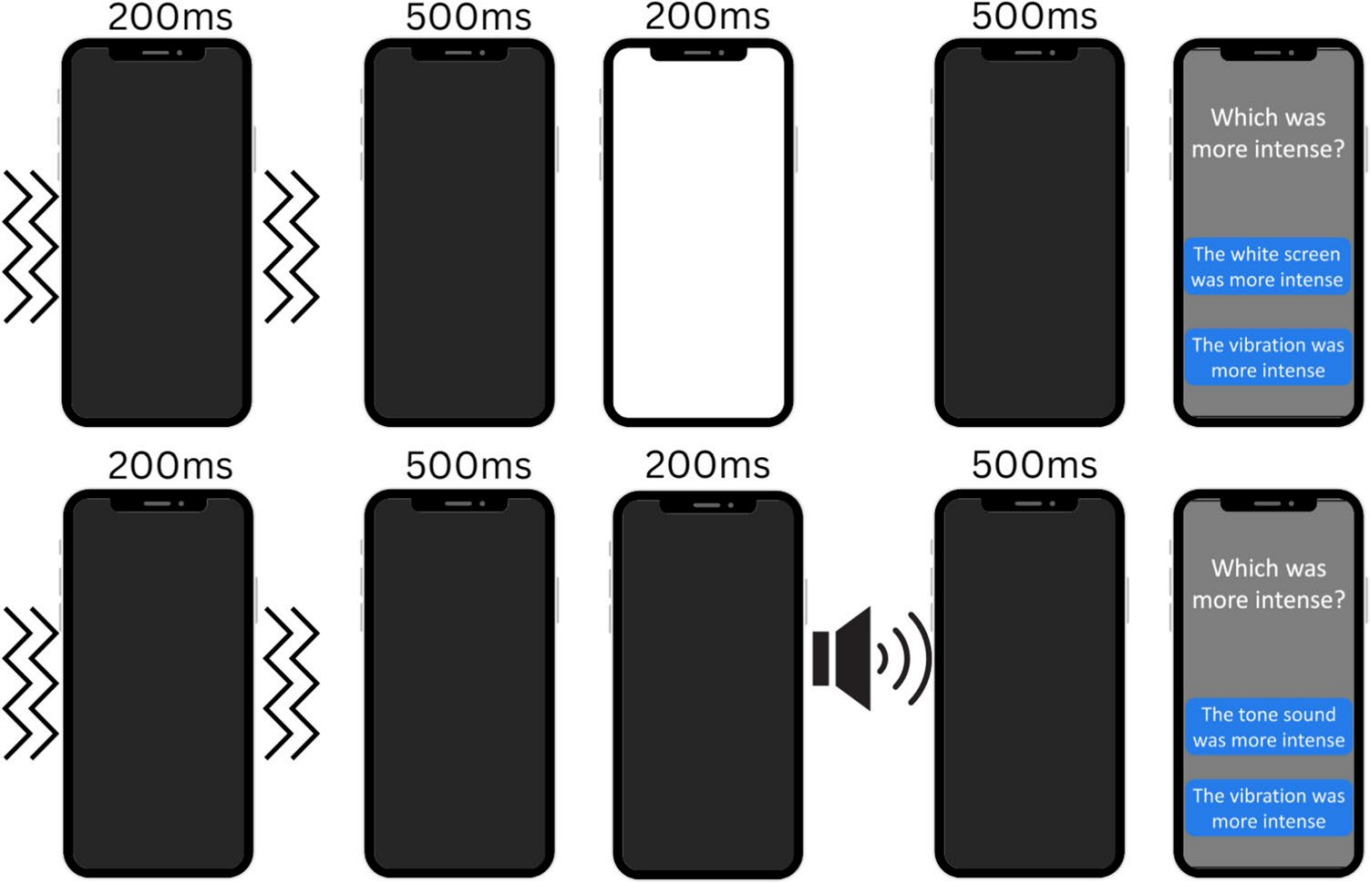
Schematic illustration of the matching task used to ensure the perceptual equivalence of stimulus intensity across modalities. On each trial, participants were presented with a black screen, which either turned white (the presentation of a visual stimulus) or remained black (during the presentation of an auditory stimulus). They were then presented with a mid-gray screen with white text, which read “Which was more intense?” Beneath this were two blue buttons reading “The vibration was more intense” and either “The white screen was more intense” (during visual matching) or “The tone sound was more intense” (during auditory matching). Participants judged the intensity of either a visual or an auditory stimulus relative to a vibration stimulus. The order of these trials was randomized, and the order of stimuli within a trial was also randomized. After each stimulus pair, participants were asked which stimulus was more intense. The visual/auditory stimulus was adjusted via a staircase using a one-up, one-down procedure until nine reversals were obtained. The average of the final six reversals was used as the stimulus intensity to use for the visual/auditory stimulus throughout the response time task

The task was presented via the participant’s mobile device, and participants were asked to hold their phone in portrait mode and respond with their right index finger.^1^ For the duration of the task, a black background was presented. Continuous brown noise was presented throughout the task to mask the sound of the vibration; the intensity of the brown noise was controlled via an initial adjustment procedure (Fig. 1). The vibration target was a 200-ms tactile vibration. The visual target occurred when the screen turned white for 200 ms. The auditory stimulus consisted of a 200-ms, 1000-Hz tone with a 20-ms ramp. The contrast of the visual target and the volume of the auditory stimulus was selected based on a perceptual matching task presented at the beginning of the task (Fig. 2) where each participant matched the perceived intensity of each target to the intensity of the vibration stimulus (see below for details).

#### Procedure

Before beginning the task, participants were instructed to remove any phone case and ensure that their device was not set to silent mode. They were advised not to wear headphones and to complete the task in a quiet environment. This instruction was intended to prevent potential audio lag, particularly from wireless headphones, which could interfere with stimulus timing.

Participants were first asked to adjust their phone volume until a sound (continuous brown noise) masked the sound from a repetitive vibration stimulus presented through their phone (Fig. 1). Vibrations were presented for 200 ms each with an inter-stimulus interval of 500 ms. Following the adjustment of the masking noise, participants were asked not to adjust the volume on their phones for the rest of the experiment.

#### Perceptual matching task

Following the initial adjustment of brown noise to mask the sound of the vibration, participants completed a perceptual matching task (Fig. 2) to align the perceived intensity of the visual and auditory stimuli with that of the tactile vibration. Continuous brown noise was played throughout this phase. The task consisted of two blocks: in one, participants adjusted the brightness of the visual stimulus; in the other, they adjusted the volume of the auditory stimulus. The order of these blocks was randomized across participants.

In each trial of the perceptual matching task, participants were presented with an auditory or visual stimulus followed by a tactile vibration stimulus, with the order of presentation (vibration first or second) randomized across participants. Stimuli were separated by a fixed 500-ms interval, and each trial was followed by a jittered inter-trial interval of 1–2 s. After the stimulus presentation, participants were asked to identify which of the two stimuli they perceived as more “intense.”

The contrast of the visual stimulus or the volume of the auditory stimulus was adjusted using a 1-up-1-down staircase procedure with a 5% step size, converging on the point of subjective equality (PSE) of intensity. Each staircase ended after eight reversals, 50 trials, or when the maximum contrast/volume was reached in the last ten trials. The PSE was calculated by averaging the final six reversal values. The contrast/volume at the PSE for the visual and auditory tasks was then applied to those stimuli in the subsequent reaction time task.

#### Reaction time task

The reaction time task was designed to replicate the Pomper et al. (2014) study as closely as possible, with the following adaptations to facilitate online remote testing. First, there were fewer trials in our study (100 in total, compared with 490 trials in Pomper et al., 2014). Second, we provided block-wise feedback to inform participants of their average speed and accuracy, to aid motivation. Third, participants were presented with text throughout to remind them how to respond “Touch the screen as fast as you can if: The screen turns white, You hear a tone, You feel a vibration, Any combination of these.”

**Fig. 3:**
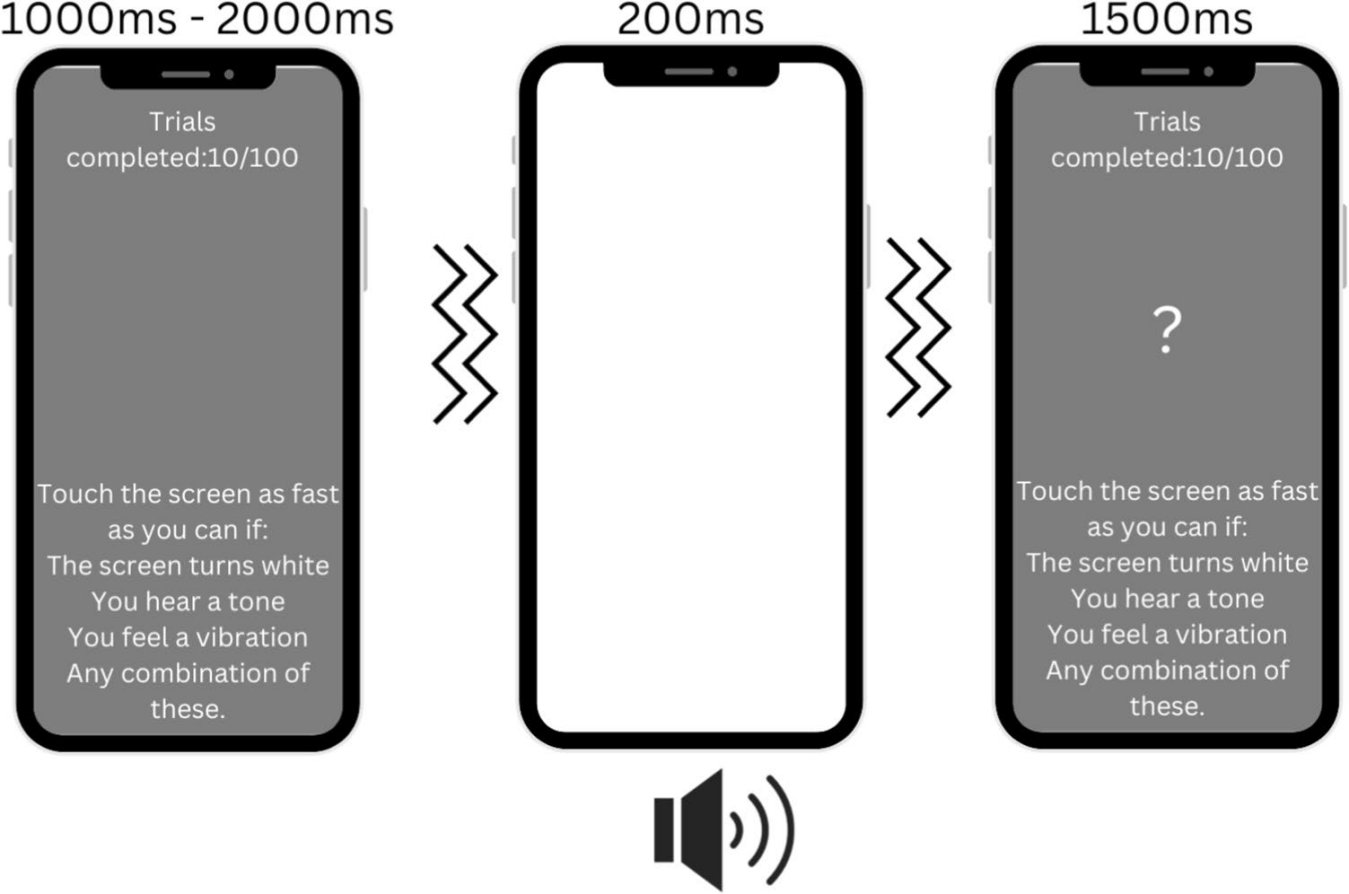
A schematic illustration of an example trial in the response time task. In this example, a trimodal stimulus was presented (white screen + tactile vibration + tone). Each trial was separated by a randomized interval of 1000–2000 ms, and the stimulus/stimuli were then presented, followed by a 1500-ms response window. Throughout the task participants were shown a mid-gray screen (which changed to white during visual stimulus presentation) with white text indicating their progress at the top of the screen (e.g. “Trials completed: 10/100)) and were reminded of the task instructions at the bottom of the screen “Touch the screen as fast as you can if: The screen turns white. You hear a tone. You feel a vibration. Any combination of these”. Following each stimulus (or blank period on no target trials), a white question mark was presented for 1500 ms in the center of the screen

Following the perceptual matching task, participants completed a reaction time task (Fig. 3) consisting of 100 trials. There were eight types of trial: 30 no target, ten visual-only, ten tactile-only, ten auditory-only, ten visuo-tactile, ten audio-tactile, ten audio-visual, and ten audio-visuo-tactile trials; No target trials were repeated 30 times to reduce the likelihood of participants repeatedly responding to stimuli. Each trial began with a fixation cross displayed for a variable duration (1–2 s), followed by the stimulus presentation (200 ms) or a 200-ms blank period for no-target trials. A question mark then appeared on screen for 1.5 s, during which participants could respond at any point, providing a total response window of 1.7 s. Block-wise feedback on average response speed was provided, with instructions to be as fast and accurate as possible. Breaks were offered after every 20 trials, and participants could choose the break duration by pressing an on-screen button to continue.

Following the reaction time task, participants were presented with a survey asking whether there was anything they wanted to report to the experimenters, e.g., whether the room became noisy, whether they received notifications during the test, or whether anything else happened that may have disrupted testing. They were also asked to report how they held their phone (with the left hand using the right finger or thumb, with the right hand using the left finger or thumb, with both hands, or in another way).

#### Analysis

Response times (RTs) less than 100 ms and over 1000 ms were removed. For each participant, RTs were also excluded if they fell more than 2.5 standard deviations from the mean for the respective condition. Analyses were performed in JASP version 0.14.1 (JASP Team, 2020). A one-way repeated measures ANOVA with seven levels was performed to assess the effect of sensory condition (visual, auditory, tactile, audio-visual, audio-tactile, visuo-tactile, audio-visual-tactile) on response time. Mauchly’s test of sphericity indicated that the assumption of sphericity was violated; therefore, Greenhouse–Geisser corrected statistics are reported.

### Results

**Fig. 4:**
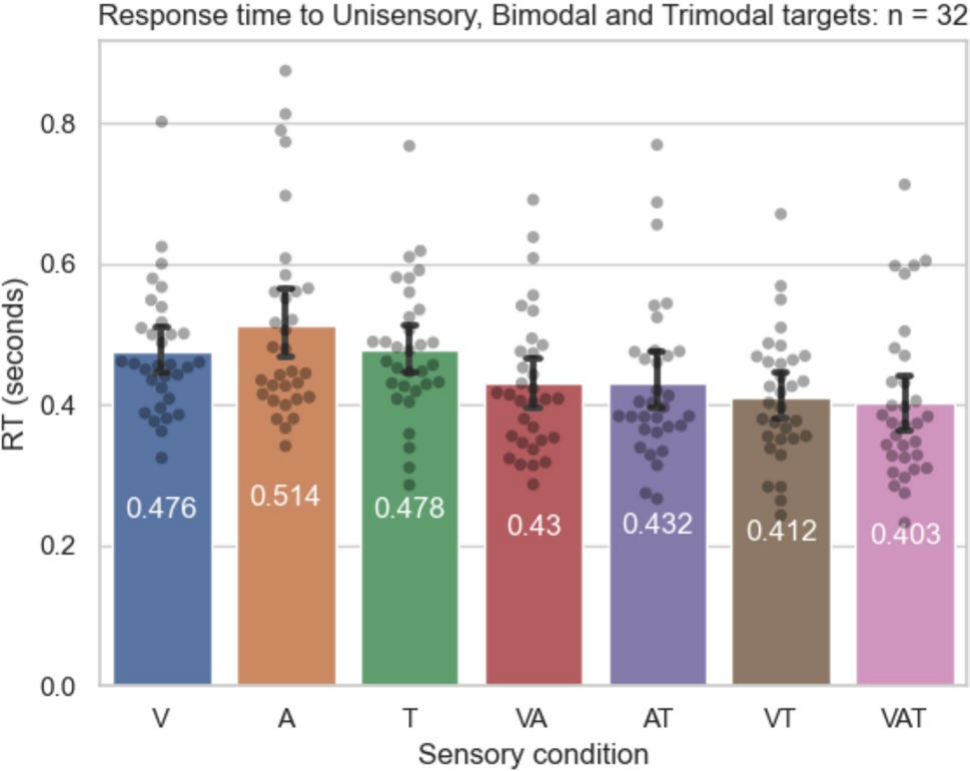
Mean response time in seconds across participants in each condition (V = visual, A = Auditory, T = Tactile, VA = Visual + Auditory, AT = Auditory + Tactile, VT = Visual + Tactile, VAT = Visual + Auditory + Tactile). Individual data points indicate the mean response time of each participant. *Bars* show the group mean, and *error bars* indicate 95% confidence interval

The mean response times to each sensory condition are shown in Table 1 and Fig. 4. There was a significant main effect of sensory condition (*F*(2.884, 89.413) = 20.118, *p* < 0.001, $\eta 2$= 0.394). Response times to unimodal auditory targets were slower than response times to unimodal visual targets (pholm = 0.032) and unimodal tactile targets (pholm = 0.043). Response times were significantly faster for all bimodal target types relative to all unimodal target types (all *p*s < 0.007). Response times were significantly faster for trimodal targets relative to all unimodal targets (all *p*s < 0.001). Response times did not significantly differ between trimodal targets and any bimodal targets (all *p*s > 0.17). For full comparisons, see Supplemental Table 1.

**Table 1:** Mean and standard deviation of response times to each sensory condition (seconds)

Sensory condition	*N*	Mean	SD	SE
Visual	32	0.476	0.093	0.016
Auditory	32	0.514	0.140	0.025
Tactile	32	0.478	0.097	0.017
Audio-visual	32	0.431	0.100	0.018
Visual-tactile	32	0.412	0.092	0.016
Auditory-tactile	32	0.432	0.113	0.020
Audio-visual-tactile	32	0.403	0.114	0.020

### Discussion

The first aim of this study was to demonstrate the feasibility of incorporating tactile vibrations into a behavioral task for remote data collection, using the redundant target effect (RTE) as a model paradigm. Our results successfully replicate a well-established multisensory effect in a fully browser-based environment, providing support for the validity of using vibration stimuli in online experiments. The task was straightforward to implement, openly accessible, and participants completed it without difficulty. Moreover, the behavioral data closely mirrored effects previously reported in controlled, lab-based settings. However, a few practical considerations emerged that are relevant for researchers adopting similar methods. For instance, although most participants followed instructions, some reported deviations in how they held their phones – highlighting the importance of designing tasks that are robust to minor variations in device handling. Additionally, although participants were asked to complete the study in a quiet setting, we could not objectively verify compliance, a common limitation in remote research. Nonetheless, the successful replication of the RTE under these uncontrolled conditions is encouraging and supports the robustness of this approach for studying multisensory processing remotely.

## Assessing the accuracy and precision of vibration stimuli

The second aim of the study was to provide hardware-based timing data for stimulus delivery via Android smartphones in the browser. These data were collected alongside a separate project exploring WebSockets as a tool for real-time communication between browser-based tasks and lab-based devices (Roberts et al., In Prep). In this manuscript, we focus specifically on the relative timing of tactile vibration delivery compared to auditory and visual stimuli, rather than the WebSocket implementation itself. These timing benchmarks serve a practical purpose: they can help researchers anticipate potential latency, such as the known delay in vibration onset due to smartphone motor ramp-up. Understanding such timing characteristics is critical when designing paradigms that rely on precise temporal alignment across sensory modalities. Ultimately, this information can inform both experimental design choices and the scope of research questions that can be effectively addressed using browser-based tactile stimuli.

### Method

PsychoPy v2024.2.4 (Peirce et al., 2019) was used to present visual, auditory, and tactile stimuli. The visual stimulus was a white square presented at the top of a black screen, the auditory stimulus was a 440-Hz pure tone, and the tactile stimulus was delivered via the mobile device’s built-in vibration using the JavaScript navigator.vibrate() method (described previously). All stimuli were presented on the Chrome browser (v133.0.6943.137) using a single Samsung a15 running Android v14 and One UI v6.1. All files used for measurements and analysis are available at https://gitlab.pavlovia.org/kalvinroberts/trimodaltiming.

Trials included a 300-ms foreperiod before stimulus presentation, in which no stimuli were presented. All stimuli were set to onset at 300 ms and lasted for 200 ms. After stimulus offset, there was a 200-ms period before the next trial commenced. In total, trials lasted 700 ms and were repeated 1000 times in blocks of 250. A WebSocket trigger (Roberts et al. In Prep) was sent at the beginning of the trial (0 ms), at stimulus onset (300 ms), and at the end of the trial (700 ms). This enabled comparison of the measured signal timing with the expected signal onset, as indicated by the WebSocket trigger.

Stimulus onset lag (accuracy) and trial-by-trial variability (precision) were assessed relative to a trigger signal. Conventional wired TTL pulses, commonly used in EEG systems, cannot be transmitted from a web browser because browsers don’t have low-level access to hardware. We therefore employed a wireless alternative, using a low-latency WebSocket connection (see Roberts et al., In Prep). The WebSocket trigger records the time at which the browser initiates stimulus presentation. We define onset lag as the mean difference between this trigger and the actual physical onset of the stimulus, and precision as the standard deviation of this difference.

**Fig. 5:**
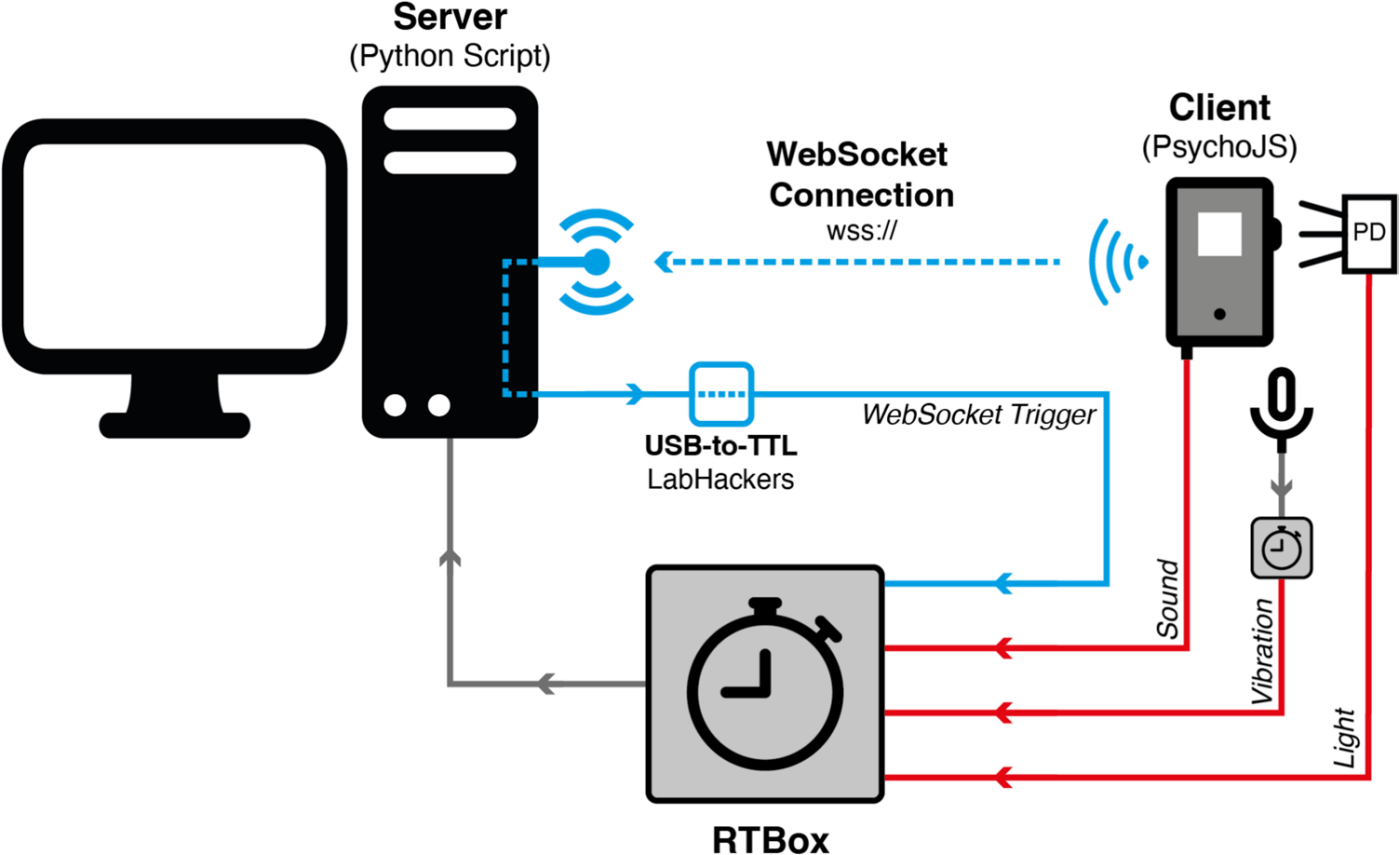
Experimental setup for stimulus presentation and timing verification. Schematic overview of the system used to deliver and measure visual, auditory, and vibration stimuli during the experiment. A PsychoJS client running on a smartphone communicated with a server over a low-latency WebSocket connection. At the start of each trial (0 ms), at stimulus onset (300 ms), and at trial offset (700 ms), the browser sent WebSocket triggers to the server, which were relayed through a LabHackers USB-to-TTL device to the primary RTBox. The smartphone delivered the visual stimulus (*white square*) detected by a photodiode (PD) connected to the RTBox light port, the auditory stimulus (440-Hz tone) recorded via a 3.5-mm jack into the RTBox sound port, and the tactile stimulus (device vibration) captured using a Blue Yeti microphone connected to a secondary RTBox. The secondary RTBox transmitted a unique TTL pulse to the primary RTBox to ensure that all signals were measured on a single, shared high-precision clock

To record three unique stimuli (visual, auditory, and tactile), two RT Boxes (Li et al., 2010) were used to give accurate timing of events where the state of inputs is monitored at an interval shorter than 0.1 ms (Fig. 5). Visual stimulus latencies were measured using a photodiode placed at the centre of the white square. The photodiode was connected to the light port on the primary RT Box. Auditory stimulus latencies were measured using a 3.5-mm jack connected to the sound port of the primary RT Box. Tactile stimulus latencies were measured using a BLUE Yeti microphone attached directly to the mobile device. The microphone was connected using a 3.5-mm jack to a secondary RT Box sound port (individual RT Boxes include only one sound port). The sound input is directly connected to a TTL output port, which can then act as a unique trigger for the primary RT Box’s TTL input port. This results in the stimulus latencies being measured on the same precise clock. To ensure the accurate recording of WebSocket timings, the system uses a Python script running on the server (available via the GitLab project for this study). This script’s sole function is to wait for a trigger signal sent from the experiment client (Pavlovia/PsychoJS) and immediately translate that signal into an electrical event. It does this by activating unique pins on the LabHackers USB2TTL device, which then sends a clean TTL input pulse directly to the primary RTBox. This allows us to detect unique TTL triggers for trial onset, stimulus onset, and trial offset. The microphone on the secondary RT Box is connected to a fourth TTL trigger on the primary RT Box.

#### Ramp onset

Because the vibration stimulus’s event marking process involved concurrent audio recordings, we were able to directly compare the recorded audio signal to the event markers associated with each vibration stimulus. This allowed us to assess the timing lag and precision of the event markers themselves. Given the ramped onset of the vibration stimuli, we expected the event marker timestamps to be delayed relative to the true physical onset. To quantify this lag, audio recordings from each session were analysed (Fig. 7a). Auditory stimulus onsets were detected automatically using a convolutional neural network (CNNOnsetProcessor; madmom library for Python; Böck et al., 2016; Schlüter & Böck, 2014), followed by peak detection (OnsetPeakPickingProcessor; madmom library) to identify precise onset timings. Initial visual inspection revealed that, although these functions reliably captured true onsets, they also occasionally detected spurious events, typically where the signal amplitude was already high. To improve onset detection accuracy, the root mean square (RMS) envelope of each recording was computed (using Librosa; McFee et al., 2024), with a frame length of 2048 samples and a hop length of 512 samples. The envelope was normalized, and any CNN-identified onset that corresponded to an RMS amplitude exceeding 0.01 was excluded as spurious. Event markers recorded by the RT Box were then matched to their nearest CNN-detected onset. The difference between each marker timestamp and the corresponding audio-defined onset was calculated to estimate the marker lag. These lag measurements were compiled across all sessions into a consolidated dataset. Finally, to assess whether the ramped nature of the vibration onset contributed to observed visual-vibration asynchronies, we performed a linear regression analysis. A significant slope in this regression would indicate that the delayed vibration onset influenced the measured asynchrony between visual and vibration stimuli.

#### Power analysis

To assess how variability in stimulus onset affects sensitivity in typical RT experiments, we performed a Monte Carlo power analysis using the LATER (Linear Approach to Threshold with Ergodic Rate) model (Noorani & Carpenter, 2016). In this framework, each trial’s decision rate *v* is drawn from a participant-specific normal distribution (truncated at zero) with mean ${\mu }_{i}$ and standard deviation ${\sigma }_{i}$; response times are then computed as $RT=1/v$. We introduced stimulus onset variability by adding zero-mean Gaussian “onset noise” (SD = 0 ms vs. 10 ms) to each RT sample. To capture realistic individual differences, each participant’s baseline drift-rate mean ${\mu }_{i}$ was sampled from $N({\mu }_{base}=2.5, {\sigma }_{\mu }=0.3)$ and variability ${\sigma }_{i}$ from $\left|N({\sigma }_{base}=0.4, {\sigma }_{\sigma }=0.1)\right|$. In the “experimental” condition, we decreased ${\mu }_{i}$ by an effect size Δμ to model slower evidence accumulation. For each simulated dataset (20 participants × 100 trials per condition), we compared mean RTs across conditions using a two-sided Wilcoxon signed-rank test. Repeating this 10,000 times per Δμ and onset-noise level yielded empirical power curves (the proportion of simulations with *p* < .05). Finally, we interpolated these curves to find the smallest Δμ achieving 80% power and translated that drift-rate difference back into an approximate RT change $\frac{1}{{\mu }_{base}}-\frac{1}{{\mu }_{base}-\Delta \mu }$ for intuitive interpretation.

### Results

**Fig. 6:**
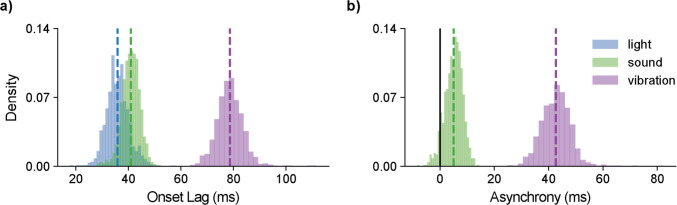
Onset lag and asynchrony for trimodal stimuli. **a)** Onset lag of visual, auditory, and haptic stimuli, where 0 ms represents the stimulus onset trigger (WebSocket signal). **b)** Asynchrony of auditory and vibration stimuli compared to the visual stimulus onset (set to 0 ms). *Dashed lines* represent means of the distributions; in subplot *b,* the *solid line* represents perfect synchrony

**Fig. 7:**
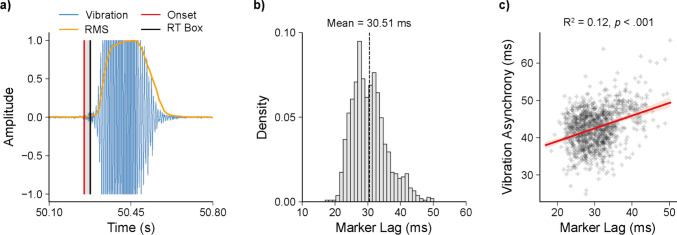
Analysis of ramp onset for vibration stimuli. **a)** Example waveform illustrating the recorded vibration stimulus (*blue*), with onsets identified by a convolutional neural network (red) and event markers recorded by the RT Box (*black*). The marker lag (*grey shaded area*) between these two onset measurements quantifies the marker's timing accuracy. The envelope of the waveform, calculated using root mean square (RMS, *yellow*), filters out spurious low-amplitude onsets to improve onset detection reliability. **b)** Distribution of marker lag values, indicating an average lag of 30.46 ms from the neural-network-defined onset. **c)** Scatterplot demonstrating a positive correlation (*R*^2^ = 0.12, *p* < .001) between marker lag and visual-vibration asynchrony, showing that marker timing variability partly explains differences in multimodal synchrony

Relative to the WebSocket trigger signal, the visual stimulus was presented with a lag of 35.9 ms and a precision of 4.3 ms (Fig. 6a, Table 2). Similarly, the auditory stimulus was presented with a lag of 40.9 ms and a precision of 3.4 ms. Tactile stimuli were presented after a longer lag of 78.6 ms at a precision of 5.1 ms. Of note, the larger lag in the tactile stimulation may be attributable to the ramp period in vibration, and the microphone not detecting vibration stimuli until a certain amplitude was surpassed. Since the microphone used to detect the vibrations was also recording during data collection, we were able to inspect the waveform of the recorded vibrations (Fig. 7a). On average, an initial ramp period before the triggering of the RT Box in these waveforms lasted 30.46 ms (SD: 5.28 ms; Fig. 7b). We conducted a simple linear regression to examine whether this ramp period predicts the asynchrony from the visual onset (Fig. 7c). The overall model was significant, *F*(1, 978) = 135.3, *p* < .001, and explained 12.2% of the variance in asynchrony (*R*^2^ = .122). The slope for ramp time was positive and significant (b_1_ = 0.342, SE = 0.029, t(978) = 11.63, *p* < .001, 95% CI [0.284, 0.400]) and so was the intercept (b_0_ = 32.23, SE = 0.91, *t*(978) = 35.46, *p* < .001, 95% CI [30.45, 34.01]). This intercept indicates that the model predicts an asynchrony of 32.23 ms even when the ramp period is 0 ms, suggesting that there is still an approximate 30 ms asynchrony when accounting for ramping of the vibration stimulus. This ramping is an inherent feature of vibration stimulus presentation, and is therefore an important factor for researchers to consider when designing experiments involving vibration. For example, one strategy might be to cue the vibration slightly earlier to ensure that its peak amplitude aligns more precisely with a visual stimulus (Table 3).

**Table 2:** Stimulus onset (lag). Time in ms of measures from photodiode (light), auditory input (sound), and microphone input (vibration) measured via the RTbox

Event	Mean	Std Dev	Min	25%	50%	75%	Max
Light	35.9	4.3	17.8	33.2	35.8	38.5	51.5
Sound	40.9	3.4	23.3	38.8	41.0	43.2	52.5
Vibration	78.6	5.1	56.1	75.5	78.5	81.6	111.7

Timings are relative to the expected stimulus onset of 0 ms. Measurements are based on 987 measured events, the mean and standard deviation of onset lags are shown, and percentages indicate raw data percentiles.

**Table 3:** Asynchrony. Time in ms of sound and vibration measurements relative to the visual stimulus. Measurements are based on 987 measured events. Percentages indicate raw data percentiles

Event	Mean	Std Dev	Min	25%	50%	75%	Max
Sound	5.0	3.2	– 8.4	2.9	5.3	7.3	14.5
Vibration	42.7	5.4	24.8	39.1	42.9	46.0	82.3

When conducting studies where response time relative to a stimulus is the dependent variable**,** a lag in stimulus onset can often be accounted for by subtracting a constant lag value from response times (where response times are calculated relative to a trigger signal), a low precision of onsets makes this calculation less reliable. Browser precision is worse compared to lab-based alternatives where visual and auditory onset precision have previously been recorded at 0.35 ms and 0.96 ms, respectively (Bridges et al., 2020). Nevertheless, even in browsers this appears to be a relatively good degree of precision. Standard deviations under 10 ms for both lags and asynchronies create a relatively stable experimental environment, as long as the expected effect size is large enough to stand out from the noise in signal onset. To assess how onset variability affects statistical sensitivity in typical RT studies, we used our Monte Carlo framework to determine the effect size (Δμ) required for 80% power. When no onset noise was added, a drift‐rate increment of Δμ = 0.0421 (≈ 6.6 ms in RT space) was sufficient to reach 80% power. Introducing 10 ms of onset variability raised the required effect size only marginally to Δμ = 0.0424 (≈ 6.7 ms). These findings indicate that the natural trial‐to‐trial variability in decision times overwhelmingly dominates the impact of a 10-ms uncertainty in stimulus onset.

Additionally, we can interpret the timing as the asynchrony between the stimuli. In this case, we can ignore the WebSocket trigger and set our latencies relative to the onset of the visual stimulus (captured using a photodiode). Previous browser-based audio-visual synchrony recorded on a Windows 10 device using Chrome and PsychoPy was reported as 65.32 ms with a precision of 3.01 ms (Bridges et al., 2020). That is, auditory stimuli were detected approximately 65 ms after the visual stimuli. Interestingly, the audio-visual synchrony on the mobile device was substantially better at 5 ms, but precision was slightly worse at 3.2 ms (Fig. 6b). The vibration stimulus lagged the visual onset by a larger 42.7 ms with a precision of 5.4 ms.

### Discussion

This study aimed to evaluate the feasibility and validity of delivering tactile vibration stimuli through smartphone web browsers for behavioral research. We adopted a two-pronged approach. First, we developed and deployed an online behavioral experiment that successfully replicated the redundant target effect (RTE) using smartphone-delivered tactile stimuli. Our paradigm illustrates how to integrate vibration stimuli into existing experiment builder frameworks, thus providing an easy-to-use solution for research. Second, we provided benchmark data on the timing accuracy and precision of vibration stimuli, comparing them with those of visual and auditory stimuli presented via the browser.

To recap our core findings: first, we successfully replicated the redundant target effect (RTE) in our sample, with effect sizes comparable to those reported in in-lab studies (Pomper et al., 2014). Our paradigm was novel not only in being delivered remotely via smartphone web browsers and in providing a means for researchers to implement this method in existing experiment builder frameworks, but also in the adaptations required to support this format. Key adaptations the reader should consider include the following. First, if the researcher is interested in isolating the effects of auditory stimuli and vibration stimuli, then the sound produced from the vibration stimulus must be masked. In the current project, we utilized brown noise and asked participants to adjust the volume of the noise until it masked the sound of a pulsing vibration. Secondly, researchers might wish to consider the inclusion of a brief perceptual matching task to approximate the intensity of visual, auditory, and tactile stimuli. This procedure is beneficial not only for remote testing, where physical calibration is not possible, but also may allow the researcher to control for individual differences in sensory function and environmental conditions (for example, by ensuring stimuli are equally as perceivable across age groups, or across individuals partaking in differing background noise). While lab-based studies typically employ longer psychophysical procedures (both for ensuring that continuous noise masks the detection of vibration sounds and to match the perceptual intensity of stimuli), we opted for a shorter version to minimize participant dropout in the online setting. Future research could usefully validate this method by comparing thresholds obtained through longer, in-lab, staircase procedures with those from brief, remote tasks.

A third adaptation to our behavioral task was the reduction in the number of trials in the response time task – 100 in total, compared to 490 in Pomper et al. (2014). Despite this reduction, we still observed a clear RTE, suggesting that online paradigms may compensate for fewer trials with larger sample sizes. A remotely deployable RTE task thus holds promise as a valuable tool for the research community, particularly given the relevance of RTE effects in clinical populations where in-person testing may be impractical. This approach may offer a scalable and accessible means of assessing multisensory integration in such groups.

Our second key finding concerns the accuracy and precision of delivering tactile vibration stimuli via smartphone web browsers. While prior studies have evaluated the timing of visual and auditory stimuli in browser-based experiments, few have focused specifically on smartphone delivery, and, to our knowledge, none have assessed vibration stimuli delivered via the browser (e.g., Inuggi et al., 2024) provide measures from a bespoke native Android app, but not for browser-based vibration delivery. Moreover, previous research has typically measured timing accuracy by comparing stimuli to one another (e.g., visual versus auditory), rather than to an absolute onset time, because there is no reliable “true zero” trigger, such as the wired TTL signals used in EEG studies.

To address this limitation, we employed a novel WebSocket-based method for marking stimulus initiation. This allowed us to measure timing not only between modalities but also relative to the expected onset of each stimulus (for further methodological details, see Roberts et al., In Prep). Using this approach, we found that both visual and auditory stimuli showed relatively consistent timing, with lags of approximately 40 ms and low variability (2–4 ms). Vibration stimuli, however, exhibited longer lags. Further analysis suggested that much of this delay stemmed from the physical ramp-up of the vibration motor, which caused a delay between stimulus initiation and detection by our microphone setup. Even after accounting for this, a residual asynchrony of around 30 ms remained (comparable to the delays observed for visual and auditory stimuli), and this too showed relatively low variability. Of particular note, our simulations suggest that within-participant-level variance remains more problematic for observing response time effects than the observed level of stimulus onset variability.

These findings offer valuable insights into the timing characteristics of browser-based stimulus delivery, particularly for tactile stimuli, and help define the types of research questions that can be reliably addressed using this methodology.

## Opportunities and caveats in browser-based vibration research

An important consideration for future research is the breadth of questions that could – and should – be explored using this simple method of delivering tactile vibration stimuli via web browsers. Based on the current study, we outline five core caveats to consider (see Table 4).

**Table 4:** Practical consideration checklist for using browser-based vibration in research

Pulse duration	We tested only 200-ms pulses. Determine whether your study requires shorter or longer pulses
Pulse interval reliability	Our setup presented a single pulse per trial; consider potential variability if presenting multiple pulses in sequence
Amplitude control	Current browser APIs do not allow manipulation of amplitude. Ensure to consider this limitation in your experimental design
Onset lag	Vibrations exhibited a ramp-up that may introduce onset delays; account for this in your design
Device and browser generalizability	We tested only Android Chrome. Reliability may differ across devices, operating systems and browsers, so verify performance on target platforms

Firstly, in the present study, we employed single-pulse vibrations of 200-ms duration. However, by varying the temporal properties, such as pulse duration, frequency, or pattern, a wider range of experimental paradigms can be supported (for example, we created a fun example demonstrating the presentation of different songs through vibration, which can be opened on Android here https://run.pavlovia.org/lpxrh6/vibrate_playlist/ on an Android device, the code for which is accessible at https://gitlab.pavlovia.org/lpxrh6/vibrate_playlist).

For example, recent work by Seveso et al. (2026) has demonstrated the successful use of tactile vibrations delivered through smartphones for online tests of object categorisation. In their study, participants categorized objects defined by object shape, visual motion, and tactile motion (i.e., vibrations), with each object category associated with distinct visual and tactile motion cues. The experiment was conducted entirely on mobile Android devices, and the results successfully demonstrated that object categorisation improved with the addition of each sensory cue: performance was most accurate when all three cues (object shape, visual motion, and tactile motion) were present compared to single-cue conditions (Seveso et al., 2026). These findings highlight both the feasibility of using vibration-based paradigms online and more importantly their potential to enrich multisensory learning in complex experimental designs.

That said, researchers should be aware of certain limitations of the current implementation. Specifically, we did not assess the feasibility of delivering shorter-duration pulses or investigate the minimum reliable interval between successive pulses. These constraints may limit the method's suitability for tasks that require high temporal precision – such as studies probing the temporal window of multisensory integration (e.g., simultaneity judgments or the touch-induced flash illusion). Future work should establish these lower bounds to clarify the method’s applicability to such time-sensitive paradigms.

Another important consideration for multisensory paradigms is the apparent lag associated with vibration stimuli, particularly when compared to visual and auditory signals. Our analyses suggest that this lag arises primarily from the ramp-up time of vibration motors – tactile signals do not reach peak intensity immediately, even when triggered simultaneously with other modalities. While our methodology allows this delay to be measured and partially corrected, failing to account for it could have important implications for certain research questions. For instance, although a stimulus asynchrony of approximately 40 ms is typically required for conscious temporal order judgments, asynchronies as small as 20 ms can influence unconscious perceptual processes (Chassignolle et al., 2021). Without compensatory timing adjustments (e.g., cueing the vibration stimulus earlier to account for the delay), the inherent lag in browser-based vibration delivery could compromise the validity of tasks such as temporal order judgments or simultaneity detection.

An additional caveat of this method is that it does not allow for precise control over the amplitude of the vibration signal, unlike traditional laboratory setups. Consequently, we cannot rule out the possibility of variability in vibration amplitude across different devices. Furthermore, browser-based vibration delivery may not be appropriate for paradigms where amplitude is the primary variable being manipulated (e.g., Verrillo et al., 1969). Future studies would benefit from systematically evaluating the consistency of vibration amplitude within a single device, across different browsers and sessions, as well as between devices. In the present behavioral study, we addressed this limitation by implementing a perceptual matching procedure. This approach is particularly valuable in multisensory research, as it ensures that participants are baseline-corrected to their own perceptual experience, thereby mitigating the impact of potential between-device amplitude differences.

Nonetheless, despite these caveats, the current findings are promising for researchers interested in conducting remote tactile research using vibration stimuli. This method opens the door to large-scale perceptual studies involving hard-to-reach populations and offers practical advantages for multi-session training paradigms, where repeated lab visits may be burdensome. By enabling stimulus delivery through widely available consumer devices, this approach enhances accessibility and scalability in tactile and multisensory research.

### Conclusion

The present study provides a practical and accessible foundation for behavioral researchers seeking to incorporate vibration stimuli into their experimental paradigms. We demonstrate that established in-lab behavioral effects can be successfully replicated in remote, uncontrolled settings using standard web browsers and consumer smartphones. In addition, we offer benchmark data on the timing accuracy and precision of browser-based vibration delivery, along with open-source experimental code to support broader adoption.

While our findings highlight the promise of this method, we also identify important limitations. Further work is required to explore the generalizability of this method across a full range of device and browser combinations. In terms of application, this approach may be less suitable for studies requiring very brief stimulus durations, tightly controlled interstimulus intervals, or precise manipulation of vibration amplitude. Nevertheless, we hope that the findings, tools, and recommendations presented here will serve as a valuable resource for researchers exploring tactile and multisensory processing in both applied and theoretical contexts.

## Supplementary Information

Below is the link to the electronic supplementary material.

Supplementary file1 (DOCX 1636 kb)

## Data Availability

All data and code supporting the findings of this study are openly available on the Open Science Framework (OSF) via https://osf.io/qf847/.

## Footnotes

1 After completing the task, participants were asked how they held their phone to respond. Fifteen reported using their left hand with the right finger, 12 reported using their right hand with right thumb, one reported using the left hand with the left thumb, three reported both hands with thumbs to respond, and one did not provide an answer to this question.

## Code availability

All code used for the experiment and analysis of this study is made available on the Open Science Framework https://osf.io/qf847/.

## References

[CR1] Böck, S., Korzeniowski, F., Schlüter, J., Krebs, F., & Widmer, G. (2016). madmom: A New Python Audio and Music Signal Processing Library. *Proceedings of the 24th ACM International Conference on Multimedia*, 1174–1178. 10.1145/2964284.2973795

[CR2] Bridges, D., Pitiot, A., MacAskill, M. R., & Peirce, J. W. (2020). The timing mega-study: Comparing a range of experiment generators, both lab-based and online. *PeerJ,**8*, e9414. 10.7717/peerj.9414 33005482 10.7717/peerj.9414 PMC7512138

[CR3] Chassignolle, M., Giersch, A., & Coull, J. T. (2021). Evidence for visual temporal order processing below the threshold for conscious perception. *Cognition,**207*, 104528. 10.1016/j.cognition.2020.104528 33296792 10.1016/j.cognition.2020.104528

[CR4] De Leeuw, J. R., Gilbert, R. A., & Luchterhandt, B. (2023). jsPsych: Enabling an open-source collaborative ecosystem of behavioral experiments. *Journal of Open Source Software,**8*(85), 5351. 10.21105/joss.05351

[CR5] Giudice, N. A., Palani, H. P., Brenner, E., & Kramer, K. M. (2012). Learning non-visual graphical information using a touch-based vibro-audio interface. *Proceedings of the 14th International ACM SIGACCESS Conference on Computers and Accessibility*, 103–110. 10.1145/2384916.2384935

[CR6] Gondan, M., & Minakata, K. (2016). *A tutorial on testing the race model inequality*. 10.3758/s13414-015-1018-y 26637234

[CR7] Hadi, R., & Valenzuela, A. (2020). Good vibrations: Consumer responses to technology-mediated haptic feedback. *Journal of Consumer Research,**47*(2), 256–271. 10.1093/jcr/ucz039

[CR8] Henninger, F., Shevchenko, Y., Mertens, U. K., Kieslich, P. J., & Hilbig, B. E. (2019). *lab.js: A free, open, online study builder*. 10.31234/osf.io/fqr49 10.3758/s13428-019-01283-5 PMC9046347 34322854

[CR9] Hershenson, M. (1962). Reaction time as a measure of intersensory facilitation l. *Journal of Experimental Psychology,**63*, 289–293. 13906889 10.1037/h0039516

[CR10] Inuggi, A., Domenici, N., Tonelli, A., & Gori, M. (2024). PsySuite: An Android application designed to perform multimodal psychophysical testing. *Behavior Research Methods,**56*(8), 8308–8329. 10.3758/s13428-024-02475-4 39138734 10.3758/s13428-024-02475-4 PMC11525261

[CR11] JASP Team. (2020). *JASP (Version 0.14.1)* [Computer software].

[CR12] Killebrew, J. H., Bensmaïa, S. J., Dammann, J. F., Denchev, P., Hsiao, S. S., Craig, J. C., & Johnson, K. O. (2007). A dense array stimulator to generate arbitrary spatio-temporal tactile stimuli. *Journal of Neuroscience Methods,**161*(1), 62–74. 10.1016/j.jneumeth.2006.10.012 17134760 10.1016/j.jneumeth.2006.10.012 PMC1851669

[CR13] Kuhlmann, T., Garaizar, P., & Reips, U.-D. (2021). Smartphone sensor accuracy varies from device to device in mobile research: The case of spatial orientation. *Behavior Research Methods,**53*(1), 22–33. 10.3758/s13428-020-01404-5 32472500 10.3758/s13428-020-01404-5 PMC7880912

[CR14] Lakens, D., & Caldwell, A. R. (2021). Simulation-based power analysis for factorial analysis of variance designs. *Advances in Methods and Practices in Psychological Science,**4*(1), 2515245920951503. 10.1177/2515245920951503

[CR15] Levordashka, A., Fraser, D. S., & Gilchrist, I. D. (2023). Measuring real-time cognitive engagement in remote audiences. *Scientific Reports,**13*, 10516. 10.1038/s41598-023-37209-7 37386031 10.1038/s41598-023-37209-7 PMC10310845

[CR16] Li, X., Liang, Z., Kleiner, M., & Lu, Z.-L. (2010). RTbox: A device for highly accurate response time measurements. *Behavior Research Methods,**42*(1), 212–225. 10.3758/BRM.42.1.212 20160301 10.3758/BRM.42.1.212

[CR17] McFee, B., Matt McVicar, Daniel Faronbi, Iran Roman, Matan Gover, Stefan Balke, Scott Seyfarth, Ayoub Malek, Colin Raffel, Vincent Lostanlen, Benjamin van Niekirk, Dana Lee, Frank Cwitkowitz, Frank Zalkow, Oriol Nieto, Dan Ellis, Jack Mason, Kyungyun Lee, Bea Steers, … Waldir Pimenta. (2024). *librosa/librosa: 0.10.2* (Version 0.10.2) [Computer software]. Zenodo. 10.5281/ZENODO.4923181

[CR18] Melnikov, A., & Fette, I. (2011). *The WebSocket Protocol. Request for Comments RFC 6455*. Internet Engineering Task Force. https://datatracker.ietf.org/doc/html/rfc6455

[CR19] Miller, J. (1982). Divided attention: Evidence for coactivation with redundant signals. *Cognive Psychology,**14*(2), 247–279. 10.1016/0010-0285(82)90010-x 7083803

[CR20] Noorani, I., & Carpenter, R. H. S. (2016). The LATER model of reaction time and decision. *Neuroscience and Biobehavioral RevieWs,**64*, 229–251. 10.1016/j.neubiorev.2016.02.018 26915927 10.1016/j.neubiorev.2016.02.018

[CR21] Novich, S. D., & Eagleman, D. M. (2015). Using space and time to encode vibrotactile information: Toward an estimate of the skin’s achievable throughput. *Experimental Brain Research,**233*(10), 2777–2788. 10.1007/s00221-015-4346-1 26080756 10.1007/s00221-015-4346-1

[CR22] Nunez, V., Gordon, J., Oh-Park, M., Silvers, J., Verghese, T., Zemon, V., & Mahoney, J. R. (2025). Measuring multisensory integration in clinical settings: Comparing an established laboratory method with a novel digital health app. *Brain Sciences,**15*(6), 653. 10.3390/brainsci15060653 40563823 10.3390/brainsci15060653 PMC12191347

[CR23] Papoutsaki, A., Sangkloy, P., Laskey, J., Daskalova, N., Huang, J., & Hays, J. (2016). *WebGazer: Scalable Webcam Eye Tracking Using User Interactions*.

[CR24] Peirce, J. W., Gray, J. R., Simpson, S., MacAskill, M., Höchenberger, R., Sogo, H., Kastman, E., & Lindeløv, J. K. (2019). PsychoPy2: Experiments in behavior made easy. *Behavior Research Methods,**51*(1), 195–203. 10.3758/s13428-018-01193-y 30734206 10.3758/s13428-018-01193-y PMC6420413

[CR25] Peirce, J. W., Hirst, R. J., & MacAskill, M. R. (2022). *Building Experiments in PsychoPy.* (2nd ed.). Sage. https://us.sagepub.com/en-us/nam/building-experiments-in-psychopy/book273700

[CR26] Pomper, U., Brincker, J., Harwood, J., Prikhodko, I., & Senkowski, D. (2014). Taking a call is facilitated by the multisensory processing of smartphone vibrations, sounds, and flashes. *PLoS ONE,**9*(8), e103238. 10.1371/journal.pone.0103238 25116195 10.1371/journal.pone.0103238 PMC4130528

[CR27] Roberts, K., Hirst, R. J., Pitiot, A., & Peirce, J. W. (In Prep). Timing Matters: Using Websockets for high-precision timing in online studies. *Manuscript in Preparation*.

[CR28] Roussel, U., Fléty, E., Agon, C., Viaud-Delmon, I., & Taffou, M. (2025). Using Android smartphones to collect precise measures of reaction times to multisensory stimuli. *Sensors (Basel),**25*(19), 6072. 10.3390/s25196072 41094896 10.3390/s25196072 PMC12526932

[CR29] Schlüter, J., & Böck, S. (2014). Improved musical onset detection with convolutional neural networks. *2014 IEEE International Conference on Acoustics, Speech and Signal Processing (ICASSP)*, 6979–6983. 10.1109/ICASSP.2014.6854953

[CR30] Seveso, M. A., Hirst, R. J., O’Dowd, A., Camponogara, I., & Newell, F. N. (2026). Visual and tactile motion cues enhance the categorisation of novel object shapes. *Experimental Brain Research, 244*(48)*. *10.1007/s00221-026-07238-5 10.1007/s00221-026-07238-5 PMC12924856 41721840

[CR31] Van Der Burg, E., Olivers, C. N. L., Bronkhorst, A. W., & Theeuwes, J. (2008). Pip and pop: Nonspatial auditory signals improve spatial visual search. *Journal of Experimental Psychology. Human Perception and Performance,**34*(5), 1053–1065. 10.1037/0096-1523.34.5.1053 18823194 10.1037/0096-1523.34.5.1053

[CR32] Verrillo, R. T., Fraioli, A. J., & Smith, R. L. (1969). Sensation magnitude of vibrotactile stimuli. *Perception & Psychophysics,**6*(6), 366–372. 10.3758/BF03212793

[CR33] Woodward, K. L., Kenshalo, D. R., & Oliff, G. K. (1990). A tactile stimulation device for measuring two-point and gap discrimination thresholds in humans. *Behavior Research Methods, Instruments, & Computers,**22*(5), 440–442. 10.3758/BF03203191

