# Supplementary material for: Delivering tactile stimuli via mobile browsers: A method for remote multisensory research
Source: Behav Res Methods. 2026 Apr 8;58(4):106. doi: 10.3758/s13428-026-02995-1 (PMC13061789; doi:10.3758/s13428-026-02995-1)
Supplement: Supplementary file 1 — Supplementary file1 (DOCX 1636 kb) [file 13428_2026_2995_MOESM1_ESM.docx]

# Supplementary material

*Supplementary S1 - Summary of Within subjects effects for the effect of sensory condition on response time.*

| ***Within Subjects Effects*** | | | | | | | | | | | | | | | |
| --- | --- | --- | --- | --- | --- | --- | --- | --- | --- | --- | --- | --- | --- | --- | --- |
| ***Cases*** | | ***Sphericity Correction*** | | ***Sum of Squares*** | | ***df*** | | ***Mean Square*** | | ***F*** | | ***p*** | | ***η²*** | |
| *Sensory Condition* |  | *None* |  | *0.317* | *ᵃ* | *6.000* | *ᵃ* | *0.053* | *ᵃ* | *20.118* | *ᵃ* | *< .001* | *ᵃ* | *0.394* |  |
|  |  | *Greenhouse-Geisser* |  | *0.317* |  | *2.884* |  | *0.110* |  | *20.118* |  | *< .001* |  | *0.394* |  |
| *Residuals* |  | *None* |  | *0.489* |  | *186.000* |  | *0.003* |  |  |  |  |  |  |  |
|  |  | *Greenhouse-Geisser* |  | *0.489* |  | *89.413* |  | *0.005* |  |  |  |  |  |  |  |
|  | | | | | | | | | | | | | | | |
| *Note. Type III Sum of Squares* | | | | | | | | | | | | | | | |
| *ᵃ Mauchly's test of sphericity indicates that the assumption of sphericity is violated (p < .05).* | | | | | | | | | | | | | | | |

*Supplementary S2 – post hoc statistical comparisons of response times between statistical conditions.*

### **Post Hoc Tests**

| **Post Hoc Comparisons - Sensory Condition ( V = visual; A = auditory; T = tactile; AV = Audio-Visual; AT = Auditory-Tactile; VT = Visual-Tactile; VAT = Visual-Auditory-Tactile)** | | | | | | | | | | | | | |
| --- | --- | --- | --- | --- | --- | --- | --- | --- | --- | --- | --- | --- | --- |
|  | |  | | **Mean Difference** | | **SE** | | **t** | | **Cohen's d** | | **p_holm_** | |
| V |  | A |  | -0.038 |  | 0.013 |  | -2.949 |  | -0.349 |  | 0.032 |  |
|  |  | T |  | -0.002 |  | 0.013 |  | -0.134 |  | -0.016 |  | 1.000 |  |
|  |  | AV |  | 0.045 |  | 0.013 |  | 3.533 |  | 0.418 |  | 0.006 |  |
|  |  | VT |  | 0.064 |  | 0.013 |  | 5.003 |  | 0.592 |  | < .001 |  |
|  |  | AT |  | 0.044 |  | 0.013 |  | 3.455 |  | 0.409 |  | 0.007 |  |
|  |  | VAT |  | 0.073 |  | 0.013 |  | 5.725 |  | 0.678 |  | < .001 |  |
| A |  | T |  | 0.036 |  | 0.013 |  | 2.815 |  | 0.333 |  | 0.043 |  |
|  |  | AV |  | 0.083 |  | 0.013 |  | 6.482 |  | 0.767 |  | < .001 |  |
|  |  | VT |  | 0.102 |  | 0.013 |  | 7.953 |  | 0.941 |  | < .001 |  |
|  |  | AT |  | 0.082 |  | 0.013 |  | 6.404 |  | 0.758 |  | < .001 |  |
|  |  | VAT |  | 0.111 |  | 0.013 |  | 8.674 |  | 1.027 |  | < .001 |  |
| T |  | AV |  | 0.047 |  | 0.013 |  | 3.667 |  | 0.434 |  | 0.004 |  |
|  |  | VT |  | 0.066 |  | 0.013 |  | 5.138 |  | 0.608 |  | < .001 |  |
|  |  | AT |  | 0.046 |  | 0.013 |  | 3.589 |  | 0.425 |  | 0.005 |  |
|  |  | VAT |  | 0.075 |  | 0.013 |  | 5.859 |  | 0.694 |  | < .001 |  |
| AV |  | VT |  | 0.019 |  | 0.013 |  | 1.471 |  | 0.174 |  | 0.616 |  |
|  |  | AT |  | -9.959×10^-4^ |  | 0.013 |  | -0.078 |  | -0.009 |  | 1.000 |  |
|  |  | VAT |  | 0.028 |  | 0.013 |  | 2.193 |  | 0.260 |  | 0.177 |  |
| VT |  | AT |  | -0.020 |  | 0.013 |  | -1.549 |  | -0.183 |  | 0.616 |  |
|  |  | VAT |  | 0.009 |  | 0.013 |  | 0.722 |  | 0.085 |  | 1.000 |  |
| AT |  | VAT |  | 0.029 |  | 0.013 |  | 2.270 |  | 0.269 |  | 0.170 |  |
|  | | | | | | | | | | | | | |
| *Note.* P-value adjusted for comparing a family of 21 | | | | | | | | | | | | | |
